# The Critical Role of SIRT1 in Parkinson’s Disease: Mechanism and Therapeutic Considerations

**DOI:** 10.14336/AD.2020.0216

**Published:** 2020-12-01

**Authors:** Xuan Li, Ya Feng, Xi-Xi Wang, Daniel Truong, Yun-Cheng Wu

**Affiliations:** ^1^Department of Neurology, Shanghai General Hospital, Shanghai Jiao Tong University School of Medicine, Shanghai 200080, China.; ^2^The Truong Neurosciences Institute, Orange Coast Memorial Medical Center, Fountain Valley, CA, USA.; ^3^Department of Neurosciences and Psychiatry, University of California, Riverside, CA, USA.

**Keywords:** Parkinson's disease, SIRT1, neuroprotection, STAC, therapeutic potential

## Abstract

Silence information regulator 1 (SIRT1), a member of the sirtuin family, targets histones and many non-histone proteins and participates in various physiological functions. The enzymatic activity of SIRT1 is decreased in patients with Parkinson’s disease (PD), which may reduce their ability to resist neuronal damage caused by various neurotoxins. As far as we know, SIRT1 can induce autophagy by regulating autophagy related proteins such as AMP-activated protein kinase, light chain 3, mammalian target of rapamycin, and forkhead transcription factor 1. Furthermore, SIRT1 can regulate mitochondrial function and inhibit oxidative stress mainly by maintaining peroxisome proliferator-activated receptor-γ coactivator-1α (PGC-1α) in a deacetylated state and thus maintaining a constant level of PGC-1α. Other studies have demonstrated that SIRT1 may play a role in the pathophysiology of PD by regulating neuroinflammation. SIRT1 deacetylases nuclear factor-kappa B and thus reduces its transcriptional activity, inhibits inducible nitric oxide synthase expression, and decreases tumor necrosis factor-alpha and interleukin-6 levels. SIRT1 can also upregulate heat shock protein 70 by deacetylating heat shock factor 1 to increase the degradation of α-synuclein oligomers. Few studies have focused on the relationship between SIRT1 single nucleotide polymorphisms and PD risk, so this topic requires further research. Based on the neuroprotective effects of SIRT1 on PD, many in vitro and in vivo experiments have demonstrated that some SIRT1 activators, notably resveratrol, have potential neuroprotective effects against dopaminergic neuronal damage caused by various neurotoxins. Thus, SIRT1 plays a critical role in PD development and might be a potential target for PD therapy.

Parkinson's disease (PD) is a progressive neuro-degenerative disease of the central nervous system (CNS). The critical pathologic mechanism of PD is the progressive loss of dopaminergic (DA) neurons in the substantia nigra (SN). The cardinal clinical symptoms of PD include bradykinesia, static tremor, rigidity, and postural instability, all of which result from the reduced number of DA neurons [1, 2]. The incidence of PD among women aged 60 to 69 and those over 80 is 30/100,000 (men: 58/100,000) and 80/100,000 (men: 258/100,000), respectively. PD seriously affects the quality of life of patients and caregivers and imposes a heavy burden on society. Currently, there is a lack of effective therapies available for patients with PD, so the development of novel treatment strategies is urgently needed.

The main neuropathological hallmarks of PD are the loss of DA neurons and the presence of Lewy bodies (LB), which mainly consist of α-synuclein. However, the precise molecular mechanisms involved in the development and progression of PD remain unclear. Recently, it has been demonstrated that aging is a major risk factor for the development of PD. Previously, aging had been recognized as a strong disease modifier, but the pathway was not fully amenable for therapeutic manipulation until the discovery of sirtuins. Sirtuins have been shown to delay aging in various species, and thus the sirtuin family has attracted great attention over recent years. Silence information regulator 1 (SIRT1), a member of the sirtuin family, is a nicotinamide adenine dinucleotide (NAD)-dependent histone deacetylase [3]. The acetylation of histones involves the transfer of an acetyl group/acetyl group to a lysine residue/lysine residue at the N-terminus of histones, which decreases the positive charge of histones and weakens their interaction with negatively charged DNA. Histone acetylation facilitates the binding of transcription factors and related enzymes to DNA, thus promoting gene transcription. In contrast, histone deacetylase mediates the opposite process and inhibits gene expression. Although previous studies have demonstrated that SIRT2 to SIRT7 are involved in cell survival and the stress response, these sirtuins have not been well studied. SIRT1 is involved in various physiological processes - it activates peroxisome proliferator-activated receptor-γ coactivator-1α (PGC-1α) to exert anti-oxidative effects and protect mitochondria (against oxidative stress) [4], activates AMP-activated protein kinase (AMPK)-mediated autophagy to remove abnormal proteins [5], and deacetylates nuclear factor-kappa B (NF-κB) to inhibit neuroinflammation [6]. SIRT1 also affects the physiological functions of normal neurons, and promotes synapse formation [7]. Recent reports have shown that SIRT1 upregulation can slow the progression of Alzheimer’s disease (AD) both in vitro and in vivo [8]. Furthermore, the correlation between aging and neurodegeneration has led researchers to investigate the role of SIRT1-related pathways in PD [9, 10]. Herein, we will discuss the critical role of SIRT1 in PD and explore the feasibility of SIRT1-related medication in the treatment of PD.

## Sirtuin family and their main function

The sirtuin family comprises a group of highly conserved class III histone deacetylases. There are seven members of this family, SIRT1-SIRT7, all of which are widely distributed in cells. Each of the sirtuins has distinct enzymatic activities, sub-cellular localizations, and physiological functions. Among them, SIRT1 has the greatest homology with Sir2 in yeast. As early as the end of the last century, Sir2, a silent information regulator in yeast, was found to delay aging and prolong the lifespan of mice [11]. Similarly, mammalian SIRT1 also exhibits a lifespan-extending effect and has been extensively studied [12]. SIRT1 was initially identified as a nuclear protein, but subsequent experiments showed that it can also shuttle into the cytoplasm during neuronal differentiation, tumor progression, and apoptosis [13]. It has also been shown to regulate DNA stability, control gene expression, maintain chromosomal structure, and control cell cycle progression. SIRT2 is located in the cytoplasm and is mainly involved in cell cycle regulation. SIRT3 is expressed in the nucleus and mitochondria and plays a role in regulating cell metabolism. SIRT4 and SIRT5 are expressed in mitochondria and regulate metabolism and mitochondrial function, respectively. SIRT6 and SIRT7 are expressed in the nucleus and regulate DNA modification and rRNA transcription, respectively [14].

As mentioned above, sirtuins are class III histone deacetylases which target histones and non-histone proteins (for example, p53 and PGC-1α) and participate in many physiological functions [15]. The sub-cellular localization, enzymatic activity, and main targets of each sirtuin are shown in Table 1.

**Table 1:** Established biochemical and functional features of human sirtuins.

	Function	Sub-cellular localization	Key targets	References
SIRT1	Deacetylase	Nucleus Cytoplasm	p53, NF-κB, IL-6, PARP-1, PPAR-γ, PGC-1α, FOXO, IGF-I,AP-1, HIF-1α	[16-25]
SIRT2	Deacetylase	Cytoplasm	α-tubulin, histone H4	[26, 27]
SIRT3	Deacetylase	Mitochondria	Acetyl-CoA acetyltransferase, FOXO3, isocitrate dehydrogenase, NADH dehydrogenase, Ku70	[28-31]
SIRT4	ADP-ribosyltransferase	Mitochondria	Malonyl-CoA decarboxylase	[32]
SIRT5	Deacetylase	Mitochondria	Cytochrome c, carbamoyl phosphate synthase	[33, 34]
SIRT6	Deacetylase ADP-ribosyltransferase	Nucleus	HIF-1α, c-Myc	[35, 36]
SIRT7	Deacetylase	Nucleus	Enzymes of nucleic acid metabolism, p53	[37, 38]

## The pathological characteristics of PD and expression level of SIRT1

PD is characterized by two key pathological changes: 1) the dysfunction and loss of DA neurons accompanied by oxidative stress, mitochondrial dysfunction, and inflammatory or immune responses; and 2) the formation of LBs consisting of α-synuclein, heat shock proteins (HSPs), and ubiquitin [39]. LBs exist in the central and peripheral nervous systems, in locations within the gastrointestinal system and spinal cord [40]. Interestingly, recent studies have indicated that α-synuclein oligomers might be able to spread between cells and tissues, just like prions [41].

SIRT1 is highly expressed in neurons and glial cells in the human brain [42] and in the hippocampus and hypothalamus of the adult mouse brain [43]. The expression of SIRT1 peaks during early embryonic development and reduces over time due to aging and pathological changes [44]. In 2008, Pallàs et al. found that SIRT1 expression was significantly downregulated in rotenone-, 1-methyl-4-phenylpyridinium (MPP^+^)-, and kainite (KA)-treated mouse primary neurons. In contrast, SIRT1 expression was upregulated in the hippocampi of mice after KA treatment. After treatment with 1-methyl-4-phenyl-1, 2, 3, 6-tetrahydropyridine (MPTP), however, SIRT1 expression in the rat midbrain was downregulated [45].

Having investigated SIRT1 expression in PD cellular and animal models, the authors then explored SIRT1 expression in brain tissue from patients with PD by measuring the protein level of SIRT1 in the cingulate gyrus. In terms of clinical symptoms, these patients had been diagnosed with PD 8 to 15 years prior and did not exhibit cognitive impairment at the time of testing. The results illustrated that there was no significant difference in the expression of SIRT1 between PD patients and healthy controls [45].

At the beginning of 2017, a more in-depth investigation was conducted [46]. This study recruited three groups of people: patients with PD; those with PD dementia (PDD); and healthy volunteers (n=12 per group). The investigators collected brain tissue from the participants and explored the expression of the three SIRT1 isoforms. The results indicated that the mean protein level of 120 kDa SIRT1 in the frontal cortex was 28% lower in patients with PD than in controls (P<0.05), and there was no significant difference in SIRT1 level in the frontal cortex between patients with PDD and controls. The protein level of 80 kDa SIRT1FL in the temporal cortex was 15% higher in PD patients than in controls (P<0.01), but there was no significant difference in SIRT1FL expression in the frontal cortex, putamen, or cerebellum between patients with PD and those with PDD. The level of 75 kDa SIRT1 isoform 2 in the temporal cortex was 36% higher in patients with PD than in controls (P < 0.01) and 26% lower in patients with PDD than in controls (P < 0.01). This study also found that the enzymatic activity of SIRT1 in the frontal and temporal lobes was significantly downregulated in the PD and PDD groups compared to controls, although no significant difference was seen between the two disease groups. Due to the small number of specimens, there is a lack of research on post-mortem brain tissue from PD patients. In conclusion, these two studies showed that the expression of SIRT1 differs across anatomical sites in patients with PD. However, the enzymatic activity of SIRT1 is disturbed in patients with PD, which may make these patients particularly susceptible to neurotoxin-induced neuronal damage.

## SIRT1 reduces α-synuclein accumulation by regulating autophagy and heat shock factor 1 deacetylation level in PD

It is well established that abnormal α-synuclein aggregation in cells plays a role in the pathogenesis of PD. Preventing or reducing the production of α-synuclein and/or promoting its elimination would delay disease progression and therefore is a promising therapeutic strategy for PD. Upregulating autophagy may have therapeutic benefits in PD. Indeed, autophagy upregulation reduces abnormal α-synuclein accumulation in PD [47]. Autophagy plays an important role in cell tolerance to starvation, removal of misfolded proteins, delay of senescence, and maintenance of intracellular homeostasis [48]. In recent years, studies into neurodegenerative diseases have found that SIRT1 can reduce the degeneration of human nucleus pulposus cells by inducing autophagy [49, 50]. SIRT1 can also reduce prion protein fragment (106-126)-induced neurotoxicity by activating autophagy [51]. Our previous study found that resveratrol, a natural agonist of SIRT1, reduced the deposition of α-synuclein in PC12 cells over-expressing α-synuclein and increased the expression of the autophagy marker LC3-II [52]. Furthermore, we showed that the activation of SIRT1 by resveratrol led to the redistribution of LC3 from the nucleus into the cytoplasm, which was accompanied by an increase in LC3-II expression and the autophagic degradation of α-synuclein and p62 in DA neurons. Inhibition of SIRT1 with EX527 blocked the decrease in acetylated LC3 levels, prevented its nuclear to cytoplasmic redistribution, decreased the level of LC3-II, and led to the accumulation of α-synuclein and p62 [53].

AMPK is an extremely important energy metabolism regulator referred to as the "metabolic sensor protein" or "energy monitor" of cells, the activity of which is primarily regulated by the AMP/ATP ratio. AMPK plays a vital role in regulating protein degradation and autophagy [54]. SIRT1 activates AMPK's major kinase, liver kinase B1 [55]. At the same time, AMPK inhibitors significantly inhibit autophagy and decrease SIRT1 expression. The reciprocal regulation of SIRT1 and AMPK may be involved in the regulation of autophagy [56]. Our previous study showed that resveratrol induces the expression of SIRT1 and promotes AMPK phosphorylation and activation [57]. As a result, the SIRT1/AMPK pathway may be a potential target for PD therapy.

SIRT1 can also induce autophagy by regulating the tuberous sclerosis 2 (TSC2) mammalian target of rapamycin (mTOR)-ribosomal protein S6 kinase 1 (S6K1) pathway and promoting neuronal survival in cerebral ischemic tissue [58]. In addition, studies have reported that the deacetylation of forkhead transcription factor 1 (FOXO1) by SIRT1 plays an important role in the development of starvation-induced autophagy [59, 60]. SIRT1-mediated RelA/p65 deacetylation can also promote autophagy to some extent [61, 62]. Therefore, we speculated that SIRT1 may promote the degradation of α-synuclein by inducing autophagy, which may have important implications for preventing or delaying the progression of PD. The studies described earlier also indicate that SIRT1 plays a key role in the regulation of autophagy through multiple pathways, although which of the known or as yet unidentified pathways is most important remains unclear.

In addition to autophagy, molecular chaperones are also involved in clearing misfolded proteins. For most proteins, molecular chaperone-mediated formation of their native conformation is one of the major post-translational modifications. In addition to assisting in protein folding, molecular chaperones also stabilize the protein and maintain the polypeptide chain components in a loosely folded state for passing through the organelle membrane [63]. More specifically, molecular chaperones can recognize the hydrophobic surface of misfolded monomers and transfer them to the ubiquitin-proteasome system or promote chaperone-mediated autophagy. This process can reduce the cytotoxicity of misfolded proteins by preventing them from accumulating in the cytosol [64]. Molecular chaperones are regulated by heat shock transcription factors (HSFs) and other cofactors to effectively remove toxic protein monomers and oligomers and promote protein homeostasis [65, 66]. The structure and function of HSFs are highly conserved and exhibit a high degree of homology. Among them, HSF1 is the most representative HSFs. In addition to phosphorylation, SIRT1 deacetylation can also activate HSF1 under stress conditions, thereby maintaining HSF1 in a deacetylated, DNA-binding competent state [67, 68]. In contrast, inhibiting SIRT1 expression with small interfering RNAs inhibits the transcription of heat shock genes. Studies have shown that SIRT1 overexpression can reduce the formation of α-synuclein aggregates [46]. However, SIRT1 does not appear to directly decrease the content of α-synuclein oligomers. Rather, SIRT1 keeps HSF1 in a deacetylated, DNA-binding competent state, which prolongs the binding of HSF1 to the heat shock promoter of the heat shock protein 70 (Hsp70). This process enhances the transcription of the Hsp70, which enhances the viability of cultured cells exposed to heat shock and induces the degradation of α-synuclein oligomers [69, 70].

In addition to affecting the clearance of α-synuclein, SIRT1 can also affect the phosphorylation of α-synuclein, which may reduce the formation of α-synuclein aggregates. Singh et al. observed a low level of basal phosphor-α-synuclein accumulation which was reduced slightly by SIRT1 overexpression. However, they also observed that under conditions of oxidative stress, over-expression of SIRT1WT reduced the formation of phospho-α-synuclein aggregates and SIRT1H363Y (a catalytically inactive variant of SIRT1) showed a similar effect to control cells. Interestingly, SIRT1 was not co-localized with phospho-α-synuclein, suggesting that the effect of SIRT1 on phospho-α-synuclein aggregate formation is indirect and independent of deacetylase activity. Rather, this effect may be mediated via an elevation of cellular anti-oxidant defense mechanisms [46].

## SIRT1 regulates apoptosis in PD

In the pathogenesis of PD, cell apoptosis is an important cause of DA neuronal loss, and this may also be the ultimate common pathway leading to the loss of DA neurons. Studies have shown that SIRT1 can reduce the damage caused by neurodegenerative diseases by regulating the apoptosis of neural cells. Consequently, targeting neuronal apoptosis may be an effective strategy for the treatment of neurodegenerative diseases.

In a previous study, resveratrol significantly reversed rotenone-induced decreases in SIRT1 expression and protein kinase B (Akt) phosphorylation and exhibited an obvious neuroprotective effect against rotenone-induced neurotoxicity. Moreover, when the SIRT1/Akt1 signaling pathway was inhibited, the neuroprotective effect of resveratrol was remarkably attenuated, which implied that SIRT1 and Akt1 could mediate neuroprotection and were potential molecular targets for intervening against rotenone-induced neurotoxicity [71]. During apoptosis, p53 interacts with members of the B-cell lymphoma 2 (Bcl-2) family, including the anti-apoptotic protein Bcl. P53 also induces Bcl-2 homologous antagonist/killer oligomerization, permeabilizes the mitochondrial membrane, rapidly induces the release of cytochrome C, and activates cell apoptosis. Our previous studies showed that resveratrol protects against neurotoxicity in a SIRT1-dependent manner. We demonstrated that SIRT1 targets H3K9 histone and regulates p53 gene expression at the transcriptional level, thus inhibiting p53 gene expression to enhance neuroprotection [57]. Studies by other investigators have shown that during cell stress, SIRT1 deacetylates the C-terminal residues of the main site of p53 ubiquitination, which helps block protein degradation and stabilize p53 [72]. SIRT1 can also deacetylate Ku70 in tumor [73, 74] and retinal cells [75] to enhance DNA repair activity and chelate Bcl-2-associated X protein (Bax) in the cytoplasm to prevent apoptosis and extend cell lifespan. However, the role of the SIRT1/Ku70 pathway in the nervous system requires further elucidation.

## SIRT1 regulates mitochondrial function and oxidative stress in PD

Studies have confirmed that mitochondrial dysfunction and oxidative stress play a role in the DA toxicity known to underlie PD. Brain tissue has a high oxygen metabolism rate but lacks an effective antioxidant protective mechanism to counteract this. Recent research has shown that the increase in early oxygen free radicals in PD can still enhance the activity of the striatum antioxidant enzyme system through the body's compensatory response and thus resist the damage caused by oxygen free radicals. However, as the disease progresses, the production of free radicals accelerates, and the body's compensatory response cannot resist the damage caused by oxygen free radicals. The striatal pro-oxidant and anti-oxidant systems fall out of balance, leading to neuronal damage and apoptosis [76]. PGC-1α is a multifunctional factor that can activate many nuclear receptors and transcription factors [77]. It has been experimentally confirmed that PGC-1α reduces cell apoptosis by increasing the activity and protein levels of anti-oxidant enzymes, whereas the expression of catalase (CAT), superoxide dismutase 1 (SOD1), and SOD2 in PGC-1α knockout mice is significantly lower than that in normal mice [78].

The histone acetyltransferase complex can directly acetylate multiple lysine residues of PGC-1α, inhibiting its transcriptional activity and decreasing the anti-oxidative activity of PGC-1α [79]. In contrast, SIRT1 activation can deacetylate PGC-1α to maintain high protein levels of this factor, thereby enhancing its anti-oxidant activity [80]. Furthermore, the SIRT1 inhibitor nicotinamide can attenuate the anti-oxidative ability of wild-type cells but not PGC-1α-overexpressing cells, confirming that PGC-1α acts downstream of SIRT1. Taken together, these studies suggest that SIRT1 upregulates PGC-1α expression during oxidative stress and imply that the SIRT1/PGC-1α pathway may play an active role in the prevention and treatment of PD.

## SIRT1 regulates inflammatory responses in PD

Studies have confirmed that microglial cells can be switched from a resting state to an activated state [81]. A recent study indicated that microglial activation can affect DA neurons by enhancing oxidative stress and promoting the production of proinflammatory cytokines that initiate an inflammatory cascade [82]. Some scholars believe that neuroinflammation damages the DA neurons of the SN and promotes their apoptosis in patients with PD [83]. In contrast, other studies have shown that neuro-inflammation has a protective effect on the CNS [84]. Whether neuroinflammation is the main cause of DA neuronal loss or a secondary response to neuronal apoptosis remains to be elucidated. Nevertheless, there is no doubt that neuroinflammation plays a role in the progression of PD [85]. Recent studies have revealed that the beneficial effect of SIRT1 on PD is due in part to its ability to suppress the transcriptional ability of nuclear factor-kappa B (NF-κB) via deacetylation [86]. It was shown that resveratrol suppressed the lipopolysaccharide (LPS)-induced degradation of IκB, expression of inducible nitric oxide synthase, and phosphorylation of p38 mitogen-activated protein kinases in N9 microglial cells. Lending further support to these findings, SIRT1 deficiency in microglia contributes to age-related cognitive decline and neurodegeneration via epigenetic regulation of interleukin-1β [87]. Furthermore, Xiu Li Bi and colleagues demonstrated that resveratrol potently inhibited the production of tumor necrosis factor-alpha and nitric oxide by LPS-activated microglial cells [88]. Taken together, these results demonstrate that SIRT1 suppresses the proinflammatory responses of microglia, suggesting that SIRT1 may participate in the progression of PD by regulating neuroinflammation.

## The relationship between SIRT1 single nucleotide polymorphisms and PD

Single nucleotide polymorphisms (SNPs) are the most common type of genetic polymorphism. SNPs can be used to locate disease susceptibility genes, assess disease risk, and prognosticate long-term outcome. Based on the neuroprotective effects of SIRT1 in PD, numerous studies have been conducted to investigate whether SIRT1 SNPs are associated with an increased risk of PD. Zhang et al. showed that three novel hybrid sequence variants located in the promoter region were identified among 97 patients with sporadic PD in northern China (g.69644133C>G, g.69644213G>A, and g.69644351G>A) [89], whereas these heterozygous sequence variants were not observed in the control population. Therefore, they speculated that these three variants may alter the transcription factor locus of the SIRT1 gene promoter, reduce SIRT1 expression, and increase the risk of sporadic PD. Another study included 326 PD patients and 371 controls from southern Spain, and genotyped 41 SNPs in sirtuin genes in order to determine whether they were related to PD risk. These SNPs included Tag SNPs, coding non-synonymous SNPs, and SNPs affecting the activity of microRNA binding sites. No relationship was identified between these SNPs and PD risk. Their data also indicate that variations in sirtuin genes do not affect PD risk, at least in the population analyzed in their study [90]. One Chinese case-control study, which included 259 patients with PD and 253 healthy controls, demonstrated that the SIRT1 rs7895833?GG mutant genotype was associated with more severe anxiety symptoms (assessed using the Hamilton Anxiety Scale) [91]. SIRT1 SNPs are associated with many age-related conditions including AD [92] and longevity [93]. It is expected that future studies will explore the basis of the interaction between SIRT1 SNPs and PD risk.

## SIRT1 activators and their neuroprotective effects in PD

Resveratrol, an activator of SIRT1, is a polyphenolic compound found in a variety of plants and drugs such as grape skin and peanuts. As a multi-targeted, pleiotropic, and natural compound, resveratrol can enter the brain through the blood-brain barrier [94] and has been found to exert protective effects in a variety of neuro-degenerative diseases, including PD [95]. In vitro, resveratrol can reduce the damage and toxic effects of oxidative stress and α-synuclein (A30P) aggregation in SK-N-BE neuroblastoma cells [96]. It can also reduce DA-induced oxidative stress in SH-SY5Y cells [97] and alleviate the apoptosis of cerebellar granule cells induced by MPP^+^[98, 99]. Resveratrol attenuates MPP^+^-induced PC12 cell death through its regulation of the pro-apoptotic factor Bax and anti-apoptotic factor Bcl-2 [99]. Resveratrol can induce autophagy by promoting the expression of heme oxygenase-1, which in turn reduces rotenone-induced PC12 apoptosis [100]. Studies using animal models of PD have shown that resveratrol enhances the activity of anti-oxidant enzymes such as glutathione peroxidase, glutathione reductase, and CAT. At the same time, it downregulates the activity of thiobarbituric acid reactive substances, protein carbonyl, and phospholipase 2. Together, these processes prevent the loss of DA neurons in the MPTP mouse model of PD [101-103].

Resveratrol also has a protective effect on 6-hydroxydopamine hydrobromide (6-OHDA)-induced PD rat models through its anti-oxidant and anti-inflammatory effects [104]. In addition, the analysis of fibroblasts from two patients with early-onset PD carrying two mutations in the PARK2 gene revealed that resveratrol regulates the downstream target of PGC-1α, reduces oxidative stress, promotes mitochondrial biosynthesis, and regulates energy metabolism [105]. Our previous studies showed that resveratrol can reduce the apoptosis of SH-SY5Y cells induced by rotenone [57] and the deposition of α-synuclein in α-synuclein-overexpressing PC12 cells [52]. Furthermore, resveratrol ameliorated motor deficits and pathological changes in MPTP-treated mice, whereas inhibition of SIRT1 by EX527 partially abolished the rescue effects of resveratrol. Therefore, exploring the neuroprotective effects of resveratrol and its underlying mechanisms may provide an experimental basis for the prevention and treatment of PD.

Despite recent data demonstrating the neuro-protective effects of resveratrol, there is still some controversy over whether it could be used as an effective pharmacotherapy. It should be noted that it is unclear how much dietary resveratrol is required to achieve beneficial effects. Various resveratrol concentrations have been reported in in vitro and in vivo studies. Ultimately, 450mg/day of resveratrol has been deemed safe for a 60-kg individual [106]. However, it is important to note that the health benefits of resveratrol are not due to its anti-oxidant activity alone, but also its anti-inflammatory and neuroprotective properties. Further studies assessing other routes of administration or pharmaceutical formulations (i.e., nanoencapsulation) are required to improve the concentration of resveratrol in target tissues and allow this drug to exert its biological effects in PD.

The design of novel compounds that can directly activate SIRT1 and improve its bioavailability remains a critical goal in the field of neuropharmacology. The first sirtuin-activating compounds (STACs) were discovered in 2003. Since then, high-throughput screening and medicinal chemistry efforts have identified more than 14,000 STACs from a dozen chemical classes, including plant-derived STACs such as stilbenes (resveratrol), chalcones (butein), and flavones (quercetin) [107] and synthetic STACs including imidazothiazoles (SRT1720) [108], thiazolopyridines (STAC-2), benzimidazoles (STAC-5), and bridged ureas (STAC-9).

Many studies have also focused on the role of phenolic compounds in PD. Among the polyphenol classes, stilbenes have been shown to exert a broad range of beneficial effects [109]. Stilbenes are ubiquitous in grapes and its related products [110]. A previous study investigated the effects of three stilbenes extracted from vine stalks - a monomer (piceatannol), a dimer (ampelopsin A), and a tetramer (isohopeaphenol) - on α-synuclein fibrillation. This study demonstrated that piceatannol could inhibit α-synuclein fibrillation by forming soluble, non-toxic α-synuclein/polyphenol oligomers in neuronal PC12 cells [111].

Dozens of STACs have been tested in animal models of type 2 diabetes, aging, neurodegeneration, osteoporosis, infection, fatty liver disease, and atherosclerosis [108, 112-116]. To date, synthetic STACs are in their fifth generation, and have an in vitro potency > 1000 times higher than that of resveratrol. Therefore, new synthetic drugs show great promise in the treatment of PD, such as some new small molecule SIRT1 activators (SRT1720, SRT2014, SRT3025, SRT2183, and SRT1460). In addition to these SIRT1 activators, a recent study using optimized high throughput screening identified E6155, a piperazine 1, 4-diamide compound, as a new small molecule activator of SIRT1. Ultimately, the authors of this study concluded that E6155 could be a promising candidate for treating insulin resistance and diabetes [117]. The role of these compounds in PD is worthy of further exploration. Sirtuin activators and their clinical applications are listed in Table 2.

**Table 2:** Sirtuin activators and their clinical applications (except Parkinson’s disease).

Compound name	Clinical applications	References
Resveratrol	Cardiovascular disease, atherosclerosis, diabetes, sleep disorders, AD, cancer	Zordoky et al. [118], Agarwal et al. [119], Seyyedebrahimi et al. [120], Pennisi et al. [121], Moussa et al. [122], Banaszewska et al. [123]
SRT2104, SRT1720, SRT2183, SRT1460 E6155 P7C3 and its analog	Type 2 diabetes, ulcerative colitis, psoriasis, effect on lipid parameters Diabetes AD, ALS, traumatic brain injury, acute liver injury, ischemic stroke, optic nerve injury	Baksi et al. [124], Sands et al. [125], Krueger et al. [126], Venkatasubramanian et al.[127] Liu et al. [89] Voorhees et al.[128], Tesla et al.[129], Blaya et al. [130], Zhang et al. [131],Oku et al. [132], Wang et al. [133]

Abbreviations: AD - Alzheimer’s disease, ALS - amyotrophic lateral sclerosis

NAD-boosting molecules constitute a newer class of STACs [134]. Since 2003, studies have shown that recycling NAD from nicotinamide by upregulating the NAD remediation pathway can mimic calorie restriction and extend the lifespan of yeast [135]. Stress and calorie restriction can activate the PNC1 gene, which encodes NAD and regulates the rate-limiting steps of the NAD salvage pathway in yeast, thus increasing the activity of Sir2 [136]. In mammals, the homolog of PNC1 is nicotinamide phosphoribosyl transferase (NAMPT). As a major precursor of NAD, nicotine is first catalyzed by NAMPT to nicotinamide mononucleotide, and is then converted to NAD [137]. NAMPT acts as a rate-limiting enzyme. Therefore, the enzymes that regulate NAD levels, such as CD38, CD157, and NAMPT, may also be potential therapeutic targets worth exploring. In a previous study, NAMPT markedly protected PC12 cells against 6-OHDA-induced oxidative stress-associated cell death. The protective effect of NAMPT may be attributed to increases in glutathione levels and SOD activity and a reduction of NAD levels, all of which are consequences of increased SIRT1 activity [138]. NAMPT, NAD, and SIRT1 may therefore play a crucial role in PD and other neurodegenerative disorders. A recent study indicated that P7C3, a compound which exerts its function by allosterically activating NAMPT [139], also blocks MPTP-mediated cell death in the SN of adult mice. Dose-response studies showed that the P7C3 analog P7C3A20 inhibits cell death with even greater potency and efficacy. These researchers further demonstrated that the hippocampal pro-neurogenic efficacy of eight additional analogs of P7C3 correlates with their protective effects against MPTP-mediated neurotoxicity [140].

Naturally occurring STACs such as resveratrol and chemically unrelated synthetic STACs activate SIRT1 in vitro by lowering its peptide Michaelis constant (K_M_) and produce pharmacological changes consistent with SIRT1 activation [108, 141]. However, the theory that STACs are direct SIRT1 activators has been widely debated. As reported by Hubbard et al., resveratrol and synthetic STACs (SRT1720 and SRT2014) increased mitochondrial mass and ATP content in wild-type, but not SIRT1 knockout myoblasts. In myoblasts expressing the E230K SIRT1 mutation, the effects of STACs on mitochondrial mass and ATP levels were also blocked [142]. However, Pacholec et al. demonstrated that SRT1720, SRT2183, SRT1460, and resveratrol are not direct SIRT1 activators by conducting several biochemical assays with native substrates and biophysical studies employing nuclear magnetic resonance, surface plasmon resonance, and isothermal titration calorimetry [143]. The broad selectivity assessment against over 100 targets including receptors, enzymes, ion channels, and transporters showed that SRT1720, SRT2183, SRT1460, and resveratrol are highly promiscuous and would not serve as useful pharmacological tools for studying SIRT1 pathways. Thus, we conclude that, to date, the evidence base from clinical studies is insufficient, contradictory, and inconclusive. We therefore recommend that further clinical trials be conducted to substantiate the neuroprotective effects of STACs and their likely mechanisms of action.

## Exercise-induced neuroprotection in PD may be related to SIRT1 activation

The beneficial effects of exercise against nigral DA neuronal vulnerability and PD progression have been shown in several studies [144, 145]. Tuon et al. demonstrated that physical exercise such as treadmill and strength training promoted neuroprotection. The mechanism underlying the neuroprotective effect of exercise may involve the activation of SIRT1, which can regulate mitochondrial function and neuroinflammation via the deacetylation of NF-κB [146]. Therefore, the neuroprotective effects of physical exercise may be mediated by SIRT1 activation [147].

**Figure 1.**
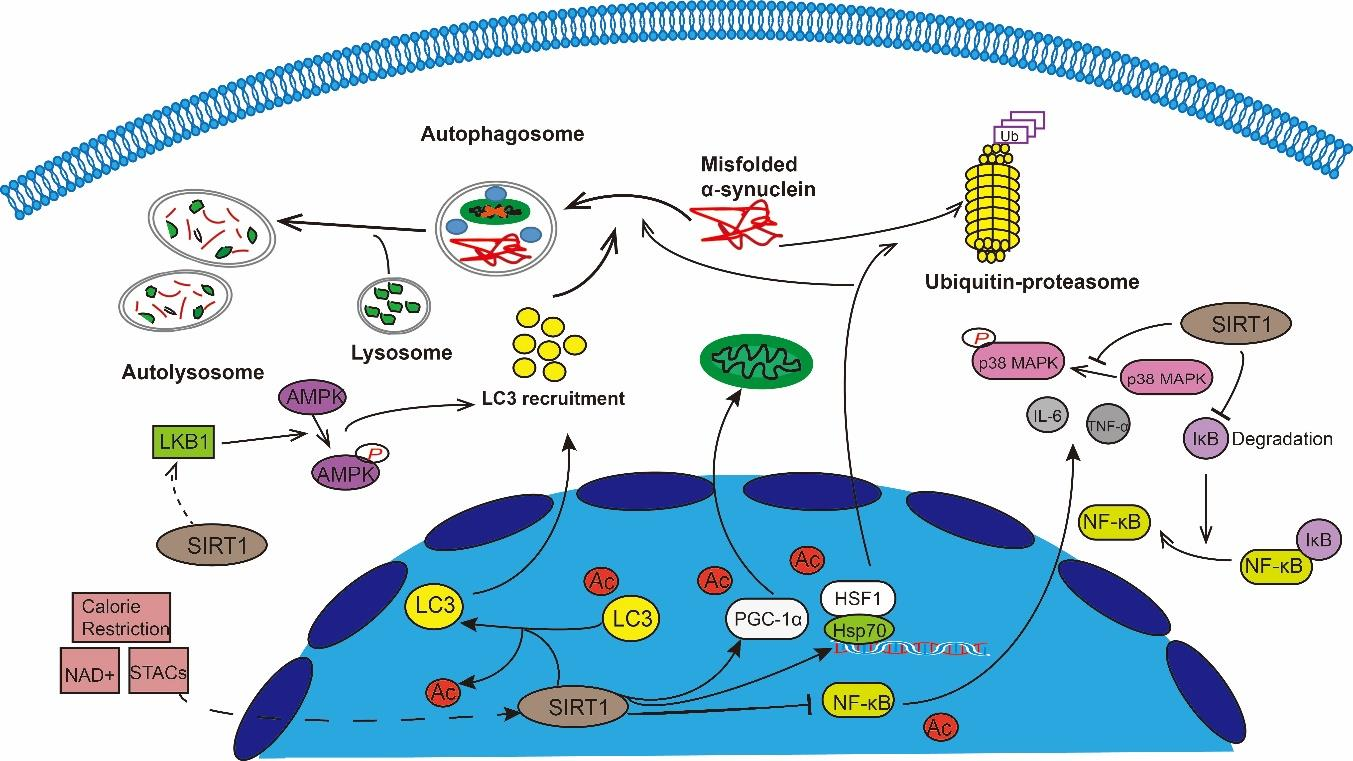
SIRT1 has been shown to participate in neuroprotective against PD through multiple mechanisms. SIRT1 can induce autophagy through regulating autophagy relevant proteins such as AMPK, Atg protein family, LC3, mTOR and FOXO1. Furthermore, SIRT1 can regulate mitochondrial function and oxidative stress mainly by keeping the deacetylated state of PGC-1α to maintain PGC-1α levels. Many experiments have demonstrated that SIRT1 may participate in the PD process through neuroinflammation. It mainly reduces NF-κB by deacetylating its transcriptional activity, inhibits iNOS expression, decreases TNF-α and IL-6 levels. SIRT1 can also up-regulate HSP70 by deacetylating HSF1 to increase the degradation of α-Synuclein oligomers.

## Conclusions

Currently, the therapeutic options available for PD are limited. The most effective treatment remains the restoration of DA neuronal function by DA supplementation. Unfortunately, however, long-term levodopa treatment is often associated with reduced efficacy and serious adverse reactions. Therefore, the identification of new therapeutic strategies is urgently needed. Aging is the major risk factor for PD, and growing evidence has shown that sirtuins are essential in delaying cellular senescence and extending organismal lifespan. SIRT1, the most extensively studied human sirtuin, is a predominantly nuclear protein that has been shown to deacetylate some non-histone targets such as p53 [57, 148], Ku70 [149, 150], peroxisome proliferator-activated receptor-γ [151, 152], PGC-1α, NF-κB, hypoxia inducible factor-1 alpha [25], and several FOXO isoforms. SIRT1 is involved in the regulation of various important cellular processes including cell proliferation, DNA repair, and apoptosis. SIRT1 has also been shown to play a role in the neuroprotective effects of PD through multiple mechanisms (Fig. 1), primarily by regulating mitochondrial function, autophagy, and neuro-inflammation.

In addition to PD, SIRT1 has been implicated in the pathophysiology of other neurodegenerative diseases. Reduced SIRT1 levels have been observed in the parietal cortex of patients with AD and an inverse correlation has been observed between SIRT1 levels and the degree of tau protein accumulation in the advanced stages of AD in humans [153]. The overexpression of SIRT1 in HEK293T cells expressing human tau led to a reduction in acetylated tau levels, while deletion of SIRT1 resulted in tau hyperacetylation. Furthermore, a glutathione S-transferase pull-down assay showed a direct interaction between SIRT1 and tau [154]. Another neurodegenerative disease in which SIRT1 has been investigated is Huntington's disease (HD). Brain-specific knockout of SIRT1 in a mouse model of HD exacerbated the pathological features of HD. SIRT1 overexpression afforded neuroprotection against HD, and this was dependent on the deacetylation of CREB-regulated transcription coactivator 1 (TORC1) by SIRT1, an interaction which increased BDNF transcription. In the presence of the mutant HD protein, the SIRT1-TORC1 interaction was inhibited, repressing BDNF transcription [155]. In addition to providing neuroprotection against neurodegenerative diseases, the activation of SIRT1 has been shown to confer protection against cerebral ischemia [156] and metabolic diseases such as obesity [157] and type 2 diabetes [158].

In summary, regulating SIRT1 expression is a potential therapeutic strategy, but the specific molecular mechanism underlying its action requires further elucidation. Furthermore, compounds found to have therapeutic benefit in other neurodegenerative diseases, such as resveratrol and chemically unrelated synthetic STACs, may hold promise for the future of PD treatment. The potential benefit of SIRT1 agonists in the treatment of PD continues to be debated and requires further exploration. Hence, large-scale, multi-center clinical trials need to be conducted to help us obtain more accurate information.
